# Prevalence and clinical implications of major and minor ANCAs in Tunisian (North African) patients with systemic lupus erythematosus

**DOI:** 10.3389/fimmu.2025.1657670

**Published:** 2025-08-22

**Authors:** Zeineb Meddeb, Houssem Abida, Dhouha Krir, Ahlem Ben Hmid, Raja Aouaidia, Cherifa Abdelkéfi, Yosra Nasri, Ines Ben Sghaier, Hayet Kebaier, Samar Samoud, Rim Goucha, Saloua B’Chir Hamzaoui, Mélika Ben Ahmed, Thara Larbi, Imen Zamali, Yousr Galai

**Affiliations:** ^1^ Faculty of Medicine of Tunis, University of Tunis El Manar, Tunis, Tunisia; ^2^ Internal Medicine Department, Mongi Slim University Hospital, La Marsa, Tunisia; ^3^ Laboratory of Genetics, Immunology, and Human Pathology, Faculty of Sciences of Tunis, University of Tunis El Manar, Tunis, Tunisia; ^4^ Clinical Immunology Department, Pasteur Institute of Tunis, Tunis, Tunisia; ^5^ Laboratory of Transmission, Control and Immunobiology of Infection, Pasteur Institute of Tunis, Tunis, Tunisia; ^6^ Nephrology Department, Mongi Slim University Hospital, La Marsa, Tunisia; ^7^ Faculty of Pharmacy of Monastir, University of Monastir, Monastir, Tunisia

**Keywords:** systemic lupus erythematosus, ANCA, lactoferrin antibodies, disease activity, organ involvement, lupus nephritis, Tunisia, North Africa

## Abstract

**Introduction:**

Anti-neutrophil cytoplasmic antibodies (ANCAs) have been reported in systemic lupus erythematosus (SLE). Their clinical significance remains unclear especially in the African populations. This study aimed to assess the prevalence, antigenic targets, and clinical correlations of ANCAs in SLE patients in a Tunisian (North African) cohort.

**Methods:**

We conducted a cross-sectional case-control study involving 30 patients with systemic lupus erythematosus (SLE) and 30 healthy controls. Blood samples were screened for antineutrophil cytoplasmic antibodies (ANCAs) using indirect immunofluorescence (IIF) (FA 1201-1005-13, Euroimmun^®^). Enzyme-linked immunosorbent assay (ELISA) (Euroimmun^®^) was performed on IIF-positive samples to assess six ANCA antigenic targets: proteinase 3, lactoferrin, myeloperoxidase, elastase, cathepsin G, and bactericidal/permeability-increasing protein (BPI). Clinical and immunological evaluations were conducted for all SLE patients at the time of the study. No ANCA- associated vasculitis-SLE overlap cases were identified.

**Results and discussion:**

ANCAs were detected in 16 of 30 SLE patients (53%) and in 1 of 30 healthy controls (3%). Among the ANCA-positive patients, nine showed reactivity to lactoferrin, while the antigenic target remained undetermined in 7 cases. The median SLEDAI-2K score at inclusion was 8 [1.75–12]. In univariate study, ANCA positivity was significantly associated with acute cutaneous manifestations (p=0.021), lupus nephritis (p=0.001), as well as use of glucocorticoids (p=0.014) and mycophenolate mofetil (p=0.009). Besides, it was associated with lower C3 (p=0.0036) and C4 (p=0.0032) titers and higher anti-dsDNA titers (p<0.0001). In multivariate analysis, ANCA positivity was correlated to anti-ds DNA (p=0.008). When comparing anti-LF positive and anti-LF negative patients, univariate analysis found an association with articular involvement (p=0.011), renal activity index (p=0.036) and ELISA titers (p=0.0004). ANCAs were frequent in our SLE cohort, with lactoferrin as the only identifiable antigenic target, unlike previous reports, which suggests a role to ethnicity and environment components. Their presence was associated with higher disease activity and more severe renal involvement.

## 1 Introduction

In daily clinical practice, biomarkers play a crucial role in connective tissue diseases, providing not only diagnostic value but also helping to predict associated clinical features and disease prognosis. In systemic lupus erythematosus (SLE), these tools are particularly useful due to the disease’s heterogeneity and the variability in its course, which can sometimes be severe (1). Beyond their well-known association with vasculitis linked to anti-neutrophil cytoplasmic antibodies (ANCA), which are inherently part of the disease’s definition, ANCA have also been detected in individuals exposed to silica, those consuming toxic substances, patients with infections or neoplasms, and more recently, those with connective tissue diseases. In fact, studies have shown that ANCA are present in the serum of 16-30% of lupus patients (2, 3). These antibodies are typically associated with renal involvement in SLE, and are most often of the anti-myeloperoxidase (MPO) type (4, 5). Less frequently, cases of overlap syndromes between SLE and ANCA-associated vasculitis (AAV) have been reported (6). In AAV, anti-MPO and anti-proteinase-3 (anti-PR3) antibodies (abs) are recognized as the major abs involved. These antibodies are essential not only for diagnosis, as they are key components of current classification criteria (7), but also for prognosis, as each is associated with distinct clinical phenotypes (8). In addition to these major antibodies, four minor target antigens have been described: lactoferrin (LF), bactericidal permeability increasing protein (BPI), elastase, and cathepsin G. However, antibodies directed against these antigens have not demonstrated specific clinical correlations in the context of vasculitis and have been shown to lack specificity for AAV (8).

The objective of our study was to explore the epidemiological, clinical, biological, immunological, and renal histological characteristics of ANCA-positive lupus patients.

## 2 Patients and methods

### 2.1 Study design

This was a cross-sectional, descriptive, and comparative study that included patients who met the 2019 American College of Rheumatology and European League Against Rheumatism (ACR/EULAR) classification criteria for SLE (9). The study included patients hospitalized or attending outpatient consultations at the Internal Medicine Department of Mongi Slim Hospital between January 2021 and December 2024 along with age- and sex-matched healthy controls (HC) consisting of volunteer health workers with no history of autoimmune diseases or inflammatory conditions or recent medication intake known to induce ANCAs. Both patients and healthy controls were aged 16 years or older. We excluded patients with active infections, malignancies, or a recent history of toxic use. Immunological assessments were carried out at the Clinical Immunology Department of the Pasteur Institute of Tunis. Healthy controls also underwent ANCA screening using the same methodology.

### 2.2 Clinical data collection

We collected demographic data and detailed SLE history for each patient. At the time of the study, a comprehensive physical examination was performed to assess symptomatic organ involvement associated with SLE. Constitutional symptoms were documented through patient interviews, with fever specifically screened by measuring body temperature. Skin and joint involvement were assessed during the physical examination. Renal involvement was evaluated through serum creatinine levels, 24-hour proteinuria assessment, and hematuria screening. Patients with impaired renal function or a 24-hour proteinuria level greater than 0.5 g/24h underwent kidney biopsy, unless contraindications to the procedure were present. Kidney biopsy specimens were analyzed by light microscopy and direct immunofluorescence. Renal lesions were classified according to the International Society of Nephrology/Renal Pathology Society revised classification for lupus nephritis (LN) of 2018 (10). Serositis was evaluated based on clinical findings such as dyspnea, chest pain, auscultation abnormalities or electrocardiogram abnormalities. Patients reporting relevant symptoms underwent chest X-ray, thoracic ultrasound, and/or transthoracic echocardiography for further assessment. Hematological involvement was screened using a complete blood count (CBC). Patients presenting with normocytic or macrocytic regenerative anemia with biological markers of hemolysis were tested for autoimmune hemolytic anemia using the direct Coombs test. Neurological involvement was screened through physical examination. In the presence of neurological symptoms, MRI imaging was performed for further evaluation. To assess disease activity, we calculated the SLE Disease Activity Index 2000 (SLEDAI-2K) at the time of sample collection, using both clinical and immunological data, as described by Gladman et al. (11).

Importantly, at the time of the study, patients were interviewed and clinically examined for signs suggestive of AAV. Renal biopsies were re-examined to assess for any features indicative of AAV-related renal involvement.

### 2.3 Immunological assessment

For each patient, 5 ml of blood was collected in tubes without anticoagulant, transported within 6 hours, and centrifuged at 3000 rpm for 15 minutes upon receipt at the Clinical Immunology Department at the Pasteur Institute in Tunis. Serum samples underwent Anti- double stranded deoxyribonucleotide acid (anti-dsDNA) screening with IIF on Crithidia Luciliae cells (Euroimmun^®^), Complement fractions (C3 and C4) (Optilite, Binding Site^®^) measurement and ANCA screening. ANCA screening was initially performed using indirect immunofluorescence (IIF) on ethanol-fixed human neutrophils (Euroimmun^®^), following the manufacturer’s instructions. Positive staining patterns were classified as cytoplasmic (c-ANCA), perinuclear (p-ANCA) or Atypical-ANCA which shows irregular cytoplasmic staining on IIF distinct from classic c-ANCA and p-ANCA patterns based on fluorescence microscopy evaluation. For samples exhibiting positive or equivocal IIF results, a comprehensive antigen-specific assessment was conducted using enzyme-linked immunosorbent assay (ELISA). The ELISA panel (Euroimmun^®^) included the following neutrophil granule antigens: MPO, PR3, LF, BPI, elastase, and cathepsin G. All assays were performed in duplicate and interpreted according to the manufacturers’ protocols and cut-off values.

### 2.4 Statistical analysis

Statistical analyses were conducted using SPSS software, version 11 (IBM Corp., Armonk, NY, USA) and GraphPad Prism 5 (Dotmatics^®^). Results of continuous quantitative variables are presented as means ± standard deviation (SD) for normally distributed data, and as medians with interquartile ranges [Q1–Q3] for non-normally distributed data. Associations between categorical variables were assessed using the Chi-square test or Fisher’s exact test, as appropriate. A comparative analysis of medians between independent groups was performed using non-parametric tests. For the multivariable analysis investigating clinical and biological factors associated with ANCA positivity and titer and lactoferrin positivity, multiple linear regressions were applied. Variables with a p-values <0.20 in univariate analyses were entered into the final multivariable model. Significance was set at p equal or inferior to 0.05.

### 2.5 Ethical considerations

Ethical approval for this study was obtained from the medical ethics committee of Mongi Slim University Hospital prior to study (34/2021). Patient confidentiality was maintained throughout the study, and all data were anonymized before analysis.

## 3 Results

Thirty SLE patients were enrolled during outpatient follow-up (n=11; 37%) and during hospitalization (n=19; 63%). They were matched with 30 HC.

Median age at the moment of study was 38 [31.7 – 44] years and twenty-eight of patients were women (93%). Patients’ clinical data at diagnosis and at study time are detailed in **Table 1**.

**Table 1:** Systemic Lupus Erythematosus patients demographic and disease characteristics.

Parameters	SLE patients at disease onset (n=30)	SLE patients at study time (n=30)
Median age	31 [22.7 – 39]	38 [31.7 – 44]
Sex-ratio (M/F)	0.07	
Clinical features		
Skin involvement	21 (70%)	11 (37%)
Malar rash	15 (50%)	10 (33%)
Oral/nasal ulcerations	5 (17%)	1 (3%)
Non scarring Alopecia	6 (20%)	5 (17%)
Photosensitivity	5 (17%)	7 (23%)
Vascular purpura	0	1 (3%)
Subacute cutaneous lupus	0	1 (3%)
Discoid lupus	3 (10%)	2 (7%)
Raynaud phenomenon	6 (20%)	6 (20%)
Renal involvement	12 (40%)	12 (40%)
Renal biopsy findings • Class III • Class III + V • Class IV • Class IV + V • Presence of crescents • Presence of necrosis • Renal biopsy not performed due to a contraindication	n=2 n=4 n=4 n=2	n=1 n=3 n=2 n=5 n=8 n=4 n=1
Articular involvement	24 (80%)	18 (60%)
Arthralgia	24 (80%)	18 (60%)
Arthritis	4 (13%)	4 (13%)
Pulmonary involvement	8 (26%)	5 (17%)
Pleurisy	7 (23%)	5 (17%)
Shrinking lung syndrome	1 (3%)	0
Cardiac involvement	4 (13%)	5 (17%)
Pericarditis	4 (13%)	4 (13%)
Myocarditis	0	1 (3%)
Hematological involvement	26 (87%)	27 (90%)
Lymphopenia	26 (87%)	27 (90%)
Leucopenia	9 (29%)	6 (20%)
Thrombopenia	5 (17%)	1 (3%)
Autoimmune Hemolytic anemia	3 (9%)	0
Neurological involvement	4 (13%)	1 (3%)
Myelitis	1 (3%)	0
Acute polyradiculoneuropathy	1 (3%)	0
Pachymeningitis	1 (3%)	0
Cognitive disorder with SEP-like lesions on MRI*	1 (3%)	1 (3%)
Cerebral venous thrombosis**	1 (3%)	0
Pancreatic involvement	1 (3%)	0

*MRI, Magnetic resonance imaging.

**Antiphospholipid syndrome ruled out by antibodies screening.

At the time of the study, renal assessment showed a median serum creatinine level at 67.5 [59.5-81] μmol/L. Median 24-hour proteinuria was 0.2 [0-2.4] g/24H. Of the patients with LN (n=12), three developed arterial hypertension and five exhibited hematuria. On pathological assessment, the median activity index was 9 [5.75-13.75], while the median chronicity index was 3.5 [1-4].

All renal biopsies showed typical histopathological findings consistent with LN (**Table 1**). One patient with LN was ANCA-negative. The renal biopsy of this patient revealed a class III plus V lupus nephritis without any necrosis or crescents. Among the ten renal biopsies performed in ANCA-positive patients, eight showed crescents and four showed necrosis.

None of our patients had clinical (ear, nose, and throat manifestations), or paraclinical features (pulmonary nodules with or without cavitation, hypereosinophilia, pure extracapillary proliferation or pauci-immune involvement on direct immunofluorescence regarding renal biopsy data) suggestive of AAV. Thus, no AAV-SLE overlap cases were identified.

At the time of the study, ANCA screening by IIF was positive in 16 patients (53%) and in 1 healthy control (3%). Immunofluorescence staining was as follows: atypical (n=10), peri- nuclear (n=5) and cytoplasmic (n=1). Antigenic analysis using ELISA for the IIF-positive patients revealed lactoferrin (LF) specificity in 9 patients, while the remaining 7 patients and the healthy control were negative. Two-thirds of patients tested positive for anti-dsDNA antibodies by IIF screening. A detailed summary of the immunological assessment is provided in **Table 2**.

**Table 2:** Systemic lupus erythematosus patients and healthy controls immunological data at study time.

Parameters	SLE patients (n=30)	Healthy controls (n=30)
Anti-dsDNA on IIF* n (%)	- 10 (33%) ++ 7 (23%) +++ 13 (43%)	NA
Median of C3 (g/L)	0.76 [0.61-1.05]	NA
Median of C4 (g/L)	0.12 [0.07-0.18]	NA
ANCA testing		
Positive IIF screening	16 (53%)	1 (3%) 2 (6%) showed Antinuclear antibodies staining pattern
Staining intensity n (%)	+ 2 (13%) ++ 11 (69%) +++ 3 (19%)	++ 1 (3%)
p-ANCA	5	1
Atypical-ANCA	10	0
C-ANCA	1	0
Positive ELISA** Screening***	9 (56%)	0
Median of ELISA titers (IU/L)	0.35 [0-1.22]	NA
ELISA antigenic target	• Proteinase-3 (PR-3) (0) • Lactoferrin (LF) (9) • Bactericidal permeability increasing protein (BPI) (0) • Elastase (0) • Cathepsin G (0)	

*IIF, Indirect immunofluorescence.

**ELISA, enzyme-linked immunosorbent assay.

***ELISA screening was conducted only to IIF positive patients NA, not applicable.

IIF intensity: - negative, +: weak, ++moderate, +++ strong.

Overall, 22 patients had active disease at the time of the study (73%), with 17 exhibiting high disease activity, as indicated by the SLEDAI-2K score.

Two patients had contraindications to hydroxychloroquine. Mycophenolate mofetil (MMF) was the most frequently prescribed disease-modifying antirheumatic drug (DMARD). A detailed overview of disease activity assessment and treatment regimens is provided in **Table 3**.

**Table 3:** Systemic lupus erythematosus activity markers and treatments.

Variable	Value
Median of SLEDAI-2k at study time	8 [1.75-12]
Rémission	8 (27%)
Low disease activity	5 (17%)
High disease activity	17 (57%)
Treatment n (%)	• Hydroxychloroquine 28 (93%) • Glucocorticoids 25 (83%) • MMF* 12 (40%) • Azathioprine 8 (27%) • Cyclophosphamide 2 (7%) • Methotrexate 1(3%) • IVIg** 1(3%)

*MMF, Mycophenolate mofetil.

**IVIg, Intravenous immunoglobulins.

At the time of the study, ANCA positivity was significantly associated with several clinical features, including cutaneous involvement (p=0.021), particularly malar rash (p=0.045) and renal involvement (p=0.001).

Regarding LN, a significant difference in both serum creatinine levels and 24-hour proteinuria was also observed between the ANCA positive and ANCA negative groups (p=0.020 and p=0.040, respectively). Anti-dsDNA showed to be significantly higher in ANCA positive patients (p=<0.0001). Additionally, C3 and C4 complement levels were significantly lower in ANCA-positive patients compared to ANCA-negative patients (p=0.0036; p=0.0032).

**Figure 1:**
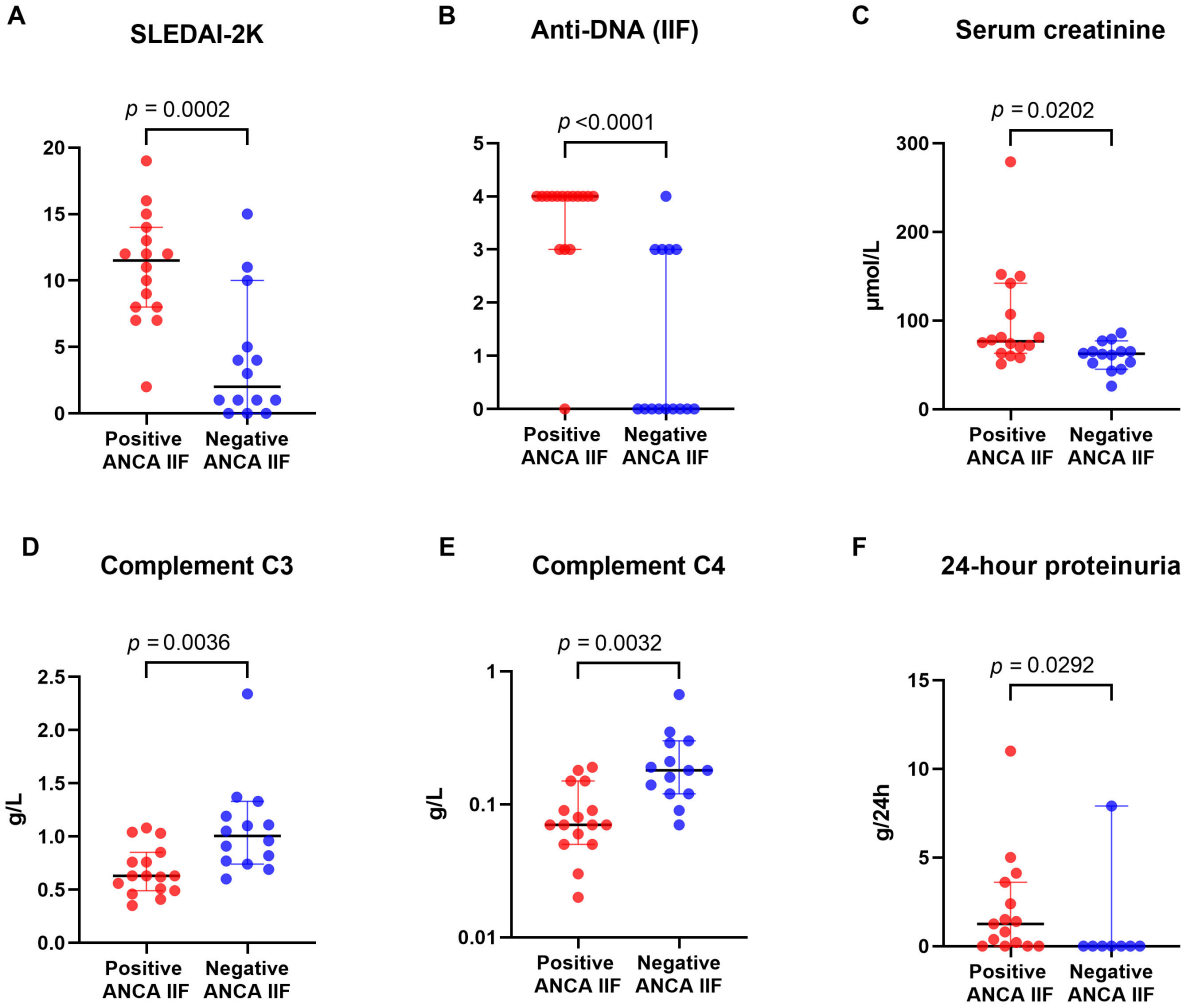
Dot plots comparing positive and negative ANCA IIF groups across six parameters: **(A)** SLEDAI-2K, **(B)** Anti-DNA (IIF), **(C)** Serum creatinine, **(D)** Complement C3, **(E)** Complement C4, and **(F)** 24-hour proteinuria. P-values indicate statistically significant differences between the two groups.

Furthermore, there was a significant difference in the SLEDAI scores between ANCA-positive and ANCA-negative patients (p=0.0002). Univariate analysis findings are summarized in **Supplementary Table 1**; **Figure 1**.

In multivariate analysis, ANCA positivity was correlated to anti-ds DNA (p=0.008). Details of multivariable analysis of both correlated factors to ANCA positivity and ELISA titers are summarized in **Tables 4**, **5**.

**Table 4:** Clinical and biological features associated with ANCA positivity by multivariable analysis.

Model	Bêta	t	p	Confidence interval 95%		Partial correlation
				LCL*	UCL**	
SLEDAI-2K	0.152	0.784	0.441	-0.023	0.050	0.099
Serum creatinemia	0.137	0.955	0.349	-0.002	0.005	0.121
c3	0.014	0.078	0.938	-0.450	0.486	0.010
Anti-dsDNA	0.512	2.917	0.008	0.042	0.247	0.370
MMF	0.239	1.435	0.164	-0.107	0.594	0.182

a. Dependant variable: ANCA positivity.

*LCL, lower confidence limit.

**UCL, upper confidence limit.

**Table 5:** Clinical and biological features associated with ANCA ELISA titers by multivariable analysis.

Model		Bêta	t	p	Confidence Interval 95%		Partial correlation
					LCL*	UCL**	
SLEDAI-2K		-0.465	-1.561	0.153	-1.561	0.153	-1.561
Serum creatinemia		0.533	2.208	0.055	2.208	0.055	2.208
c3		0.042	0.129	0.900	0.129	0.900	0.129
Anti-dsDNA		0.105	0.363	0.725	0.363	0.725	0.363
MMF		-0.097	-0.326	0.752	-0.326	0.752	-0.326

a. Dependant variable: ANCA titers (ELISA).

*LCL, lower confidence limit.

**UCL, upper confidence limit.

When comparing anti-LF positive and anti-LF negative patients, univariate analysis found an association with articular involvement (p=0.011), renal activity index (p=0.036) and ELISA titers (p=0.0004) (**Table 6**). Multivariate analysis did not find any of these associations (**Supplementary Table 2**).

**Table 6:** Univariate comparison of anti-lactoferrin positive and negative patients.

Parameters	Anti-Lactoferrin positive patients (n=9)	Anti-Lactoferrin negative patients (n=7)	p
Skin involvement	5 (55.6%)	4 (57.1%)	1
Lupus nephritis	6 (66.7%)	5 (71.4%)	1
Serum creatinemia	75 [66.5 - 146]	78 [60 - 81]	0.837
24-Hour proteinuria	1.5 [0 - 3.8]	0.82 [0.15 - 2.30]	0.677
Arthralgia	3 (33.3%)	7 (100%)	0.011
Arthritis	0	3 (42.9%)	0.063
Pleurisy	2 (22.2%)	2 (28.6%)	1
Pericarditis	4 (44.4%)	7 (100%)	0.088
Hematological involvement	3 (33.3%)	1 (14.3%)	0.585
Flares number	2 [1 - 5]	1.14 ± 0.37	0.252
ELISA titer (IU/L)	0.91 [0.43 – 1.41]	0	0.0004
C3 (g/l)	0.51 [0.435 - 0.740]	0.76 [0.63 - 1.03]	0.071
C4 (g/l)	0.070 [0.045 - 0.0120]	0.70 [0.05 - 0.18]	0.758
SLEDAI-2K	10 [8 - 13]	12 [7 - 15]	0.2183
Renal biopsy findings (n=10) - Class III - Class IV - Class V - Class IV + V - Presence of crescents - Presence of necrosis - Activity index - Chronicity index	n=6 n=1 n=2 n=1 n=3 n=5 n=4 13 [9.5 - 15] 4 [2.5 - 13.5]	n=4 n=2 n=2 n=1 n=1 n=3 n= 0 7.3 ± 2.08 2 ± 1.73	0.333 0.667 0.600 0.452 0.667 0.071 0.036 0.250

## 4 Discussion

To the best of our knowledge, this is the first study conducted in Tunisia and North Africa to evaluate ANCA positivity and its associated clinical features in SLE patients. ANCAs were detected in 53% of SLE patients and 3% of controls, with an absolute difference of 50% (95% CI: 31% to 69%), indicating a statistically significant and clinically relevant association. ELISA testing identified antigenic targets in 56% of ANCA-positive patients, with anti-lactoferrin antibodies detected in nine cases. ANCA antibodies were associated with higher disease activity, organ involvements (LN and acute cutaneous lesions), and the use of Glucocorticoids and MMF. These results are of particular interest given the limited data on ANCA profiles in North African populations, and they highlight potential immunological variations influenced by ethnicity or environmental exposures. They contribute meaningfully to the understanding of SLE immunoprofiles in underrepresented populations.

A HC group was also included to assess the presence of ANCA antibodies in the general population and revealed ANCA positivity on IIF only in one participant. Furthermore, we performed a comprehensive immunological analysis of ANCA, extending beyond the major antigens PR3 and MPO to include minor antigenic targets LF, BPI, Elastase and Cathepsin G. Finally, we ensured a standardized clinical, biological and immunological assessment to all patients at study time which allowed better data interpretation.

The frequency of ANCA antibodies in our cohort (53%) was notably higher than previously reported, as earlier studies have estimated ANCA prevalence in SLE patients to be around 30% (12).

True overlap syndromes involving both SLE and ANCA-associated vasculitis (AAV), however, appear to be rare (13), and no such overlap cases were identified in our cohort.

Regarding antigenic specificity, LF was the sole antigenic target identified in 9 out of 16 ANCA-positive patients in our cohort. This result contrasts with previous studies that identified myeloperoxidase (MPO) as the most common ANCA target in patients with SLE (4, 14). Dual specificity for both MPO and proteinase 3 (PR3) has also been reported (15). This finding suggests an impact for environmental and ethnic factors on the prevalence of antigenic specificities. In the literature, the prevalence of anti-LF antibodies in SLE patients ranges from 8.4% to 59% (16). This latter figure, which is comparable to our results, was reported in Serbian (Caucasian) patients (16). Outside of SLE, these antibodies have also been detected in approximately 10% of patients with rheumatoid arthritis and in 19% of those with systemic sclerosis (17). The predominance of anti-lactoferrin specificity in our cohort may reflect both genetic background and regional environmental exposures—such as silica, infectious triggers, or pollutants—that have been implicated in ANCA induction (18) These findings support the hypothesis that population-specific factors influence the autoantibody repertoire in SLE and contribute to its clinical heterogeneity.

Univariate analysis found an association in anti-LF positive with articular involvement (p=0.011), renal activity index (p=0.036) and ELISA titers (p=0.0004) suggesting a potential role for these antibodies as biomarkers of disease activity and organ-specific manifestations in SLE. Previous studies have reported that IgG anti-lactoferrin antibodies are associated with renal involvement (17, 19) in SLE, consistent with our results for this manifestation. A correlation with markers of renal disease activity (anti-dsDNA) was also demonstrated (18) However, the association with joint involvement appears novel, as prior literature, did not identify a significant link with arthritis (19, 20). This discrepancy may reflect differences in patient cohorts, disease activity, or assay sensitivity. This also may be attributable to the subjective nature of arthralgia, which can be experienced with varying degrees of intensity among patients. It is worth noting that this association, although statistically significant in univariate analysis (p = 0.011), did not persist in multivariate analysis. We underscore the fragility of this signal that need further mechanistic exploration in larger cohorts.

Furthermore, IgG anti-LF antibodies have also been linked to Raynaud’s phenomenon, serositis, including pericarditis and a history of thrombotic events (19, 21). These findings suggest that anti-lactoferrin antibodies may contribute to the inflammatory processes underlying SLE, potentially through neutrophil activation or immune complex formation, and highlight the need for larger studies to validate their prognostic utility.

Other ANCA subtypes, such as anti-defensin and anti-cathepsin antibodies, have been reported in SLE in a limited number of cohorts and appear to be associated with higher overall disease activity, though without clear links to specific clinical features (22). Anti-BPI antibodies, on the other hand, are rare in SLE, with most studies reporting a prevalence below 0.8% (16, 23). One exception is a single cohort that reported a prevalence of 23% (13/55) (20) but the small number of patients with this subtype precluded any meaningful conclusions about associated clinical features. Thus said, other studies didn’t find any of these differences (22, 23).

Similar to the scarce literature (23), we didn’t find any difference regarding the demographic characteristics between ANCA negative and ANCA positive SLE patients.

We found that ANCA positivity was associated with the presence of acute cutaneous SLE lesions, particularly malar rash. However, it is important to acknowledge that patients with chronic cutaneous lupus were underrepresented with only 2 patients in our cohort, likely due to their follow-up being primarily managed in dermatology clinics. The evidence regarding this association remains inconclusive. While some studies support our findings and have also reported associations with other acute cutaneous manifestations such as oral ulcers, photosensitivity, and cicatricial alopecia (3), others have not identified a significant correlation (16).

Secondly, the frequency of serositis, and particularly pericarditis, in our ANCA-positive patients did not reach statistical significance.

This association has been reported in previous studies (3, 16). In addition to pericardial involvement, some cohorts have noted a higher frequency of myocarditis among ANCA-positive patients (16).

Some other studies suggest a higher prevalence of neurosomatic manifestations in ANCA- positive patients, that we didn’t find (3).

Third, and in line with existing literature, we observed a correlation between ANCA positivity and the presence of LN. Studies from European and North American cohorts report LN prevalence among ANCA-positive SLE patients ranging from 3.69% to 16.8% (24), with a reported positive predictive value of 76.2% (16). Notably, male patients and individuals of Asian descent appear to show the highest rates of ANCA positivity in SLE (12). In our cohort, serum creatinemia and proteinuria was significantly higher in univariate analysis. In other settings, studies have demonstrated that biological presentation for LN is similar between ANCA positive and ANCA negative patients, with comparable serum creatinine, proteinuria and hematuria levels (25).

Studies have reported no meaningful differences in classes of LN between the two groups (3, 24). Other authors have emphasized the frequency of Class IV-S lupus nephritis and glomerular necrosis in the ANCA-positive group (14). Besides, a higher incidence and proportion of glomerular sclerosis (3) and a higher chronicity index (3, 25) were described in the ANCA-positive group. ANCA was an independent risk factor for poor renal outcomes in these LN patients (3).

We were unable to assess the relationship between ANCA positivity and the histopathological classification of lupus nephritis, nor between ANCA positivity and the activity and chronicity indices, since only one patient with biopsy-proven LN was ANCA- negative, which did not allow for a comparative analysis. However, the only patient with lupus nephritis and negative ANCA had neither necrosis nor crescents on renal biopsy. Moreover, among the ten renal biopsies performed in ANCA-positive patients, eight showed crescents and four showed necrosis which could supports the association between ANCA positivity and more aggressive histological findings in our LN patients.

Lastly, among ANCA-positive patients, renal involvement appears to be influenced by the ANCA subtype. As mentioned above, LN may be more frequent in the presence of anti-LF antibodies (16), as suggested by our findings. However, what is more consistently reported in the literature is that patients with MPO-associated LN tend to exhibit more severe renal involvement compared to those with PR3-associated LN [5]. Specifically, MPO-LN patients often present with more pronounced markers of renal impairment, yet show less evidence of complement activation. They are also more likely to display features of chronic kidney damage, including interstitial fibrosis and tubular atrophy. Overall, renal survival rates in MPO-LN patients appear to be lower than those observed in PR3-LN patients (4).

Overall, ANCA positivity was associated with higher disease activity, as measured by the overall SLEDAI-2K score. This finding aligns with previous studies, which have also reported a correlation between ANCA titers, their avidity levels, and the SLEDAI-2K score (19). Additionally, we observed that activity biomarkers were more significantly altered in ANCA- positive patients. Similar results have been reported in earlier studies; where ANCA-positive patients exhibited lower complement fraction levels and higher anti-dsDNA titers (3).

This study has several limitations. First, the relatively small sample size and the partially retrospective, single-center design limits the generalizability and interpretation of the results. Second, a selection bias must be acknowledged: since the majority of our patients were hospitalized (63%), the proportion of individuals with active lupus—and consequently the prevalence of ANCA positivity—may be overestimated. Third, we did not conduct longitudinal clinical and immunological follow-up, which limited our ability to assess ANCA titer kinetics and their potential long-term prognostic value. Fourth, because data on environmental exposures such as silica or medication use were not systematically collected, matching for these variables in the control group was not possible.

## 5 Conclusion

In this first North African study assessing the prevalence and impact of ANCA in the presentation of systemic lupus erythematosus (SLE), these biomarkers were found to be associated with specific organ involvement, particularly lupus nephritis (LN) and acute cutaneous lupus. Moreover, ANCA positivity was linked to more biologically active LN. While similar associations have been reported in previous studies, our findings revealed an unexpected observation: all ANCA-positive patients exhibited specificity to LF. This is in contrast to the commonly reported predominance of anti-MPO antibodies. Such a discrepancy may reflect ethnic or environmental influences unique to our population. However, these observations warrant confirmation in larger cohorts with longitudinal follow-up.

## Data Availability

The original contributions presented in the study are included in the article/**Supplementary Material**. Further inquiries can be directed to the corresponding author.

## Supplementary material

Supplementary Table 1 Comparison of ANCA-positive and ANCA-negative patients.

Supplementary Table 2 Multivariate comparison of anti-lactoferrin-positive and anti-lactoferrin-negative patients.a. Dependant variable: Lactoferrin positivity LCL: lower confidence limit. UCL: upper confidence limit.
