# Supplementary material for: Prevalence and clinical implications of major and minor ANCAs in Tunisian (North African) patients with systemic lupus erythematosus
Source: Front Immunol. 2025 Aug 22;16:1657670. doi: 10.3389/fimmu.2025.1657670 (PMC12411488; doi:10.3389/fimmu.2025.1657670)
Supplement: Supplementary Table 1 — Comparison of ANCA-positive and ANCA-negative patients. [file Table1.pdf]

SUPPLEMENTARY TABLE 1

Supplementary table 1. Comparison of ANCA-positive and ANCA-negative patients

Parameters	ANCA-positive SLE patients (n=16)	ANCA-negative SLE patients (n=14)	*p*
Age at disease onset	36 [26.2-39]	29 [19.5-40.2]	0.193
Age at diagnosis	39.5 [32.7-44]	35 [29-41]	0.377
Sex-ratio (M/F)	0.14	0	0.276
Skin involvement	9	2	0.021
Malar rash	8	2	0.045
Subacute lupus	1	0	0.533
Discoid lupus	1	1	0.724
Arthralgia	10	8	0.529
Arthritis	3	1	0.352
Serositis	6	1	0.061
Pericarditis	4	0	0.066
Pleurisy	4	1	0.209
Lupus nephritis	11	1	0.001
Activity index	9 [8-14]	5	0.2
Chronicity index	4 [1-4]	1	0.4
Arterial hypertension	4	0	0.066
Serum creatinemia (µmol/l)	76.5 [64.7-133.2]	62.5 [50.2-68]	0.020
24-hour proteinuria (g/24h)	0.2 [0 – 2.4]	0 [0]. NB: 1 outlier : 7.6 g/24	0.029

Leucopenia<br>(< 4 x 10^3/μL)	4	2	0.395
Thrombopenia<br>(< 150 x 10^3/μL)	1	0	0.533
C3 (g/L)	0.63 [0.49-0.82]	1 [0.76-1.22]	0.0036
C4 (g/L)	0.07 [0.05-0.13]	0.18 [0.12-0.29]	0.0032
Anti-dsDNA	15	5	<0.0001
SLEDAI-2k	11.5 [8-13.7]	2 [0.75-6.25]	0.0002
Flares number	1 [1-2]	1 [0-2]	0.101
Glucocorticoids	16	9	0.014
Methotrexate	0	1	0.467
Azathioprine	4	4	0.574
Mycophenolate mofetil (MMF)	10	2	0.009
Cyclophosphamide	0	2	0.209
