# Supplementary material for: Prevalence and clinical implications of major and minor ANCAs in Tunisian (North African) patients with systemic lupus erythematosus
Source: Front Immunol. 2025 Aug 22;16:1657670. doi: 10.3389/fimmu.2025.1657670 (PMC12411488; doi:10.3389/fimmu.2025.1657670)
Supplement: Supplementary Table 2 — Multivariate comparison of anti-lactoferrin-positive and anti-lactoferrin-negative patients.a. Dependant variable: Lactoferrin positivity LCL: lower confidence limit. UCL: upper confidence limit. [file Table2.pdf]

SUPPLEMENTARY TABLE 2

Supplementary table 2. Multivariate comparison of anti-lactoferrin-positive and anti-lactoferrin-negative patients

Model	Bêta	t	*p*	Confidence Interval 95%		Partial correlation
				LCL*	UCL**	
ELISA titers	0.110	0.075	0.952	-16.678	16.875	0.017
Activity index	0.429	0.447	0.732	-1.699	1.823	0.099
Chronicity index	-0.139	-0.118	0.925	-1.077	1.057	-0.026
Presence of necrosis	0.602	0.649	0.634	-10.835	12.001	0.144
C3	-0.123	-0.245	0.847	-14.097	13.564	-0.054
SLEDAI-2K	-0.225	-0.878	0.541	-0.520	0.453	-0.194

a. Dependant variable: Lactoferrin positivity

*LCL: lower confidence limit

**UCL: upper confidence limit
